# A Comparison among Score Systems for Discharging Patients from Recovery Rooms: A Narrative Review

**DOI:** 10.3390/nursrep14040205

**Published:** 2024-10-06

**Authors:** Khadija El Aoufy, Carolina Forciniti, Yari Longobucco, Alberto Lucchini, Ilaria Mangli, Camilla Elena Magi, Enrico Bulleri, Cristian Fusi, Paolo Iovino, Pasquale Iozzo, Nicoletta Rizzato, Laura Rasero, Stefano Bambi

**Affiliations:** 1 Department of Health Sciences, University of Florence, 50134 Florence, Italy; khadija.elaoufy@unifi.it (K.E.A.); camillaelena.magi@unifi.it (C.E.M.); paolo.iovino@unifi.it (P.I.); l.rasero@unifi.it (L.R.); stefano.bambi@unifi.it (S.B.); 2 Medical and Surgical Intensive Care Unit, Careggi University Hospital, 50134 Florence, Italy; forcinitic@aou-careggi.toscana.it; 3 UOS Terapia Intensiva Generale e UOSD Emergenza Intraospedaliera e Trauma Team, Fondazione IRCCS San Gerardo dei Tintori, 20900 Monza, Italy; alberto.lucchini@unimib.it; 4 Urological Ward, Careggi University Hospital, 50134 Florence, Italy; ilaria.mangli@edu.unifi.it; 5 Intensive Care Unit, Department of Anesthesiology, Emergency and Intensive Care Medicine, Ente Ospedaliero Cantonale (EOC), CH-6500 Lugano, Switzerland; enrico.bulleri@eoc.ch (E.B.); cristian.fusi@eoc.ch (C.F.); 6 Emergency Department, Azienda Ospedaliera Universitaria Policlinico Paolo Giaccone, 90127 Palermo, Italy; pasquale.iozzo@policlinico.pa.it; 7 Operating Room, Bellaria Hospital, AUSL Bologna, 40139 Bologna, Italy; nicolettarizzato76@gmail.com

**Keywords:** recovery room, post-anesthesia care unit, discharge, postoperative patient, score system, vital signs, patient safety

## Abstract

Introduction: The recovery room (RR) is a hospital area where patients are monitored in the early postoperative period before being transferred to the surgical ward or other specialized units. The utilization of scores in the RR context facilitates the assignment of patients to the appropriate ward and directs necessary monitoring. Some scoring systems allow nurses to select patients who can be discharged directly to their homes. Aim and methods: The aim of this narrative review was to describe and compare the scoring systems employed to discharge postoperative patients from RR, with a focus on item characteristics. Results: Nine scoring systems were identified and discussed: the “Aldrete Score System” and its modified version, the “Respiration, Energy, Alertness, Circulation, Temperature Score”, the “Post Anesthetic Discharge Scoring System”, the “White and Song Score”, the “Readiness for Discharge Assessment Tool”, the “Anesthesia and Perioperative Medicine Service Checklist”, the “Post-Anesthetic Care Tool”, the “Post-operative Quality Recovery Scale”, and the “Discerning Post Anesthesia Readiness for Transition” instrument. Discussion and conclusions: To obtain a comprehensive overview, the items included in the scoring systems were compared. Despite the availability of guidelines for patients’ discharge readiness from the RR, there is no universally recommended scoring system. Next-generation scores must be improved to ease their use, minimize errors, and increase safety. The main goals of the scores included in this narrative review were to be simple to use, feasible, intuitive, comprehensive, and flexible. However, these goals frequently conflict because patient assessment takes time, and a smart and comprehensive score may not consider some clinical parameters that may be crucial for the discharge decision. Therefore, further research should be conducted on this topic.

## 1. Introduction

The post-anesthesia care unit (PACU), also known as the recovery room (RR), is a hospital area where postoperative patients are monitored until their recovery of consciousness and circulatory stability [1,2] before being transferred to the surgical ward, or, if needed, other specialized units, such as the intensive care unit (ICU) or high-dependency unit (HDU).

All surgical specialties and adult or pediatric patients can be cared for in the RR/PACU [3]. The primary aims of the RR are: to monitor vital signs; assess neuromuscular, metabolic, and renal functions; manage the effects of anesthesia and surgical procedures during the immediate postoperative period [4].

The main postanesthesia complications include nausea and vomiting, shivering, hypothermia, altered level of consciousness, urinary dysfunction, dizziness, cardiovascular complications, and respiratory dysfunction [5,6,7].

Tiret et al. found that out of 103 patients who underwent anesthesia, 58% of complications occurred during the anesthesia phase, while the remaining 42% occurred within the first 24 h after surgery. Among these events, 75% occurred within the initial five hours post-surgery [5]. Furthermore, postoperative mortality was higher than intraoperative mortality [5].

The RR allows for the admission of patients from the operating room for varying amounts of time, based on clinical characteristics, the type of surgery, anesthesia, and sedation. A key objective of the RR is to provide postanesthetic care not only for surgical procedures but also for diagnostic and therapeutic interventions that may require sedation or anesthesia, such as endoscopy, computed tomography (CT), and magnetic resonance imaging (MRI) [8].

The RR ensures that patients receive appropriate monitoring and care as they recover from the effects of anesthesia or sedation before transitioning to further care or discharge.

Indeed, the length of stay (LOS) in the RR can vary and is determined by the clinical condition, type of surgery, anesthesia, or sedation performed.

Some studies reported an average LOS of 66.62 min [9], whereas others reported a median LOS of 117 min [10], with experience reporting an LOS of less than 20 min [11].

In some cases, patients are discharged directly from the RR to their homes after an appropriate monitoring period.

Effective discharge planning benefits greatly from interdisciplinary teamwork, which includes not only nurses and anesthesiologists but also surgeons and other healthcare professionals.

To enhance safe and successful recovery after surgery, nurses working in the RR should have received specialized training in postoperative care [12]. Nurses are crucial in assessing patient readiness for discharge, managing recovery, and ensuring that patients meet the necessary criteria, while anesthesiologists are instrumental in evaluating patients’ immediate postanesthesia recovery, which directly influences discharge decisions.

Nurses with different skills, education, and training provide care in the RR. Also, the roles and tasks that can be performed by RR nurses differ throughout the world. Two areas have been identified as important in the education and training of RR nurses: training in intensive care and competencies to provide basic and advanced life support [13]. In the study by Hegarty et al., most decisions to discharge patients from the RR were made by nurses, highlighting the decisional autonomy of skilled nurses that is supported by several scoring systems for the RR discharge of patients [14]. These scoring systems use a defined set of clinical measures to determine patient severity and predict outcomes [15].

Indeed, a scoring system is a structured method of quantifying a patient’s clinical status or health-related outcomes. They use various criteria—such as symptoms, clinical signs, laboratory results, or patient behaviors—to assign a numerical score, which helps assess a specific condition or risk level. Scoring systems are commonly used in healthcare to standardize assessments, guide treatment decisions, predict outcomes, and monitor progress.

To perform postoperative patient monitoring and surveillance, the RR requires an adequate nurse-to-patient (N/P) ratio, which varies according to the policies of local hospitals. A review of guidelines from various European and non-European countries showed that there is no common shared indication of the N/P ratio [4]. In countries such as France and Australia, the N/P ratio is typically one to three, but it can become one to one in the case of unconscious patients. The American Society of Anesthesiologists (ASA) has urged local authorities to make decisions, while other institutions have adapted the N/P ratio based on the RR’s operating hours and patients’ surveillance times [4].

The utilization of scores in the RR context facilitates the assignment of patients to the appropriate ward following surgery and directs necessary monitoring. Since the establishment of RRs, many scores have been used to assess patients [16,17,18,19,20,21,22,23,24]. The first scores described in the scientific literature were developed to monitor and discharge patients from the RR to the most appropriate ward. Later, newer-validated scoring systems were introduced to select patients who could be discharged directly to their homes. This breakthrough was made possible by progress in anesthesia and surgical techniques, as well as the availability of increasingly sophisticated monitoring systems. In the US, when an anesthesiologist is not available, nurses are provided with the authority to discharge patients from the RR autonomously, using specific scores with predetermined cut-offs and pending satisfaction with specific criteria [4]. A systematic review published in 2013 aimed to identify the essential components of an effective and feasible RR. The authors included eight studies, yet with limited high-quality research and a high risk of bias, with some key recommendations that specific variables must be assessed before discharging patients from RR, such as pain, conscious state, blood pressure, and nausea and vomiting [25]. More recently, a review focused on the assessment tools used in the postoperative discharge scoring criteria after outpatient anesthesia, resulting in 14 score-based tools [26].

Thus, the aim of the present narrative review is to describe and compare the scoring systems used to discharge postoperative patients from the RR to surgical wards or home, with a particular focus on their item characteristics and their state of validity and reliability.

## 2. Materials and Methods

A narrative review methodology was adopted to provide an in-depth understanding of the complex issue of the scoring systems used to discharge postoperative patients from the RR [27].

Nevertheless, this typology of review is also recommended when the purpose is to promote continuing education as an update on specific topics [28].

This narrative review followed the SHC-21-17-Reporting Checklist (Supplementary Materials Table S1) [29].

### 2.1. Search Strategy

A comprehensive literature search was carried out on PubMed/MEDLINE, Cumulative Index to Nursing and Allied Health Literature (CINAHL), and Embase with no time restrictions. Targeted Internet searching using Google Scholar was also examined for additional studies of interest, including the first five pages of records.

The following terms “recovery room” and “post-anesthesia care unit”, and its abbreviation “PACU”, were selected for the search strategy. Moreover, a text–word search was used rather than a subject search (i.e., a Medical Subject Headings terms search), as suggested by previous evidence, despite the risk of losing specificity [30]. The adapted search strategy for each database is provided in the Supplementary Materials (File S2).

The present narrative review was conducted in September 2023.

### 2.2. Eligibility Criteria

Eligibility criteria were defined prior to the database search as follows: (a) articles in English or Italian languages, (b) quantitative studies, (c) abstracts, and (d) full-text availability.

### 2.3. Study Selection and Data Extraction

Titles and abstracts of the retrieved records were independently screened for eligibility by two reviewers (I.M. and C.F.). The identified full texts were then retrieved and independently assessed for eligibility criteria by the same two reviewers. In case of disagreement, a third author (S.B.) served as a tiebreaker.

For the included studies, characteristics were extracted and synthetized as follows: name of the score, number of items, whether the score is point-based or dichotomous, the score range, the adopted cut-off score for discharging from RR, whether the tool is designed to discharge at home or to another hospital ward, whether the tool is validated, and strengths/weaknesses of the tool.

## 3. Results

**Figure 1:**
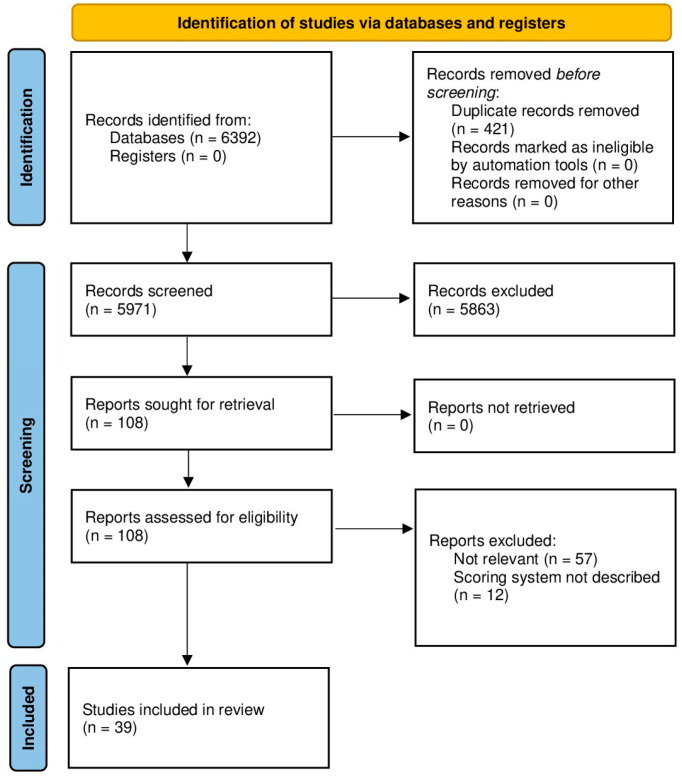
Flow chart of the study selection process.

A total of 6379 records were identified, plus 13 from Google Scholar (total records retrieved 6392). After the removal of 421 duplicates, 5971 records were screened for title and abstract, 108 of which were selected for full-text evaluation. A total of 39 studies were included in the narrative review (Figure 1).

### 3.1. Characteristics of the Available Recovery Room Scores

The characteristics of the scores used to monitor and discharge postoperative patients from the RR are shown in Table 1. Moreover, a comparison of the included tools is shown in Table 2, as the criteria for patient discharge from the RR have evolved over time. The development of new scores over the years indicates the need to improve the RR discharge system in terms of its speed and safety.

#### 3.1.1. Aldrete Score System and Modified Aldrete Score System (MASS)

The Aldrete Score System, developed by Aldrete and Kroulin, was the first scoring system designed for patients discharged from the RR [16]. It was inspired by the Apgar score used for newborn assessment and aimed to provide objective information about patients’ clinical condition in the RR. It comprises five items: respiration, skin color, level of consciousness, circulation, and muscle activity. Monitoring is recommended periodically during the patient’s stay in the RR—every hour for the first two hours, and beyond three hours if needed. Each item score ranged from 0 to 2, with a total score of 0–10. A score of 10 indicated the best possible clinical condition, while a score of 9 indicated that the patient could safely be discharged from the RR to the surgical ward.

A modified version of the Aldrete score, known as the Modified Aldrete Score System (MASS), was introduced in 1995 by Aldrete [17]. To reflect monitoring advances in technology, the item “skin color” was changed to “oxygen saturation” [17]. Additionally, the monitoring intervals were also adjusted to include more frequent assessments: at admission, and at 5, 15, 30, 45, and 60 minutes, as well as at discharge. Despite these changes, the cut-off score for safe discharge from the RR remained unchanged [17].

With the rise of same-day surgery procedures, Aldrete developed an additional scoring system called the Same-Day Surgery score, aimed to expedite and safely manage patient discharge from the RR to their homes [17]. Five new items were added to the MASS: wound appearance, pain, ambulation, early feeding, and urinary output. Each item is scored from 0 to 2, with a total score ranging from 0 to 20. The minimum discharge score for same-day surgery was established at 18.5.

Aldrete also developed the Expanded Post-Anesthetic Recovery Score for Ambulatory Patients (PARSAP), which includes five additional indices for discharge criteria from Phase II recovery. These indices included dressing, pain, ambulation, fasting/feeding, and urine output. The modifications involved removing the oxygen saturation category and rewording other categories [31].

#### 3.1.2. Respiration, Energy, Alertness, Circulation, Temperature (REACT) Score

In 1984, Fraulin and Murphy developed the REACT (Respiration, Energy, Alertness, Circulation, Temperature) score as an alternative scoring system for patient assessment in RR [18]. This scoring system evaluates five key items: respiration, energy, alertness, circulation, and temperature, with each item scored from 0 to 2, resulting in a total score ranging from 0 to 10 [18]. The “Respiration” item assesses the need for ventilatory support; “Energy” evaluates limb mobility; “Alertness” reflects the level of consciousness; “Circulation” assesses blood pressure. Body temperature is also measured as part of the evaluation.

A score of 10 indicates full recovery from anesthesia and readiness for transfer to the hospital ward. The REACT score is not recommended for use in cases of acute clinical changes such as bleeding, arrhythmias, or asphyxia [18]. It was designed to offer a more objective approach compared to the Aldrete Score System.

Moreover, by replacing the “skin color” item from the Aldrete score with body temperature, the REACT score addresses potential challenges related to assessing skin color, which can vary with skin tones and lighting conditions [18].

#### 3.1.3. The Post-Anesthetic Discharge Scoring System (PADSS)

The Post-Anesthetic Discharge Scoring System (PADSS) was developed to facilitate patients’ discharge from the RR directly to their homes [19]. The PADSS evaluates several clinical discharge criteria, including stable vital signs (i.e., blood pressure, heart rate, respiratory rate, and body temperature) pain levels, patient wakefulness and orientation, the absence of nausea and vomiting, steady gait during ambulation, minimal bleeding, and fluid intake and output. This scoring system was developed to assist physicians and nurses in evaluating patient conditions in the Ambulatory Surgery Unit at Toronto General Hospital. However, its reliability and validity have not been fully evaluated [19].

Each item yields a score ranging from 0 to 2, resulting in a maximum scale score of 10. A cut-off of 9 allows for the possible discharge of patients at home, but this is not the only criterion needed for discharge. Indeed, the presence of an adult accompanying a patient home is required [19].

A Modified Post-Anesthetic Discharge Scoring System (MPADSS) was later proposed. Changes in the MPADSS included removing the intake/output item, separating the pain and nausea/vomiting items into distinct items, and eliminating the assessment of the consciousness level [20]. These modifications were made due to the concerns about the intake/output criterion [35,36]; specifically, waiting for the patient to empty their bladder could delay discharge without substantial benefit. Thus, this criterion is now recommended only for patients at a high risk of urinary retention [35]. It is recommended that patients first meet the Aldrete criteria before being assessed and discharged using the PADSS or MPADSS [20]. The fundamental requirements for patient discharge remain unchanged [20].

#### 3.1.4. White and Song Score (Fast-Track Scoring System)

In 1999, the “Fast-track Scoring System”, also known as the White and Song Score, was introduced for ambulatory surgery under general anesthesia. This system facilitates the direct discharge of patients from the operating room to Phase II of postanesthetic recovery, thereby bypassing the RR [21,37]. Postanesthetic recovery is divided into three phases: immediate, intermediate, and late recovery [38,39]. The intermediate recovery phase, which takes place in the surgical ward or day surgery area, involves less-intensive monitoring and begins once coordination, motor skills, and physiological vital signs have normalized [38,39].

The duration of the intermediate phase can range from one to six hours, depending on the patient’s condition [22]. The White and Song score was designed to enable direct discharge from the operating room by integrating criteria from the MASS and adding considerations for major anesthesia-related side effects such as nausea, vomiting, and pain [21,37].

The score ranges from 0 to 14, with a minimum score of 12 required for discharge and no item scoring less than one. This scoring system aims to reduce costs and nursing workloads during the recovery phase [21]. However, not all patients are suitable for fast-tracking according to validated protocols, and some may still need to stay in the RR [39].

Although initially applied to ambulatory surgery, the White and Song score has increasingly been used in combination with the Aldrete score as a criterion for discharging all postoperative patients from the RR to the hospital wards.

#### 3.1.5. Readiness for Discharge Assessment Tool (RDAT)

The Readiness for Discharge Assessment Tool (RDAT) was developed in response to the lack of widely accepted standards for discharge from the RR [23]. The tool emerged from a literature review and was influenced by a scoring system that demonstrated the effectiveness of using predetermined discharge criteria to reduce hospital stay length without compromising patient safety [40]. This study indicated that nurse-led discharge based on these criteria was both quicker and safe compared to traditional physician-led discharge [40]. The RDAT identified key criteria for assessment, including activity, respiration, pulse, blood pressure, oxygen saturation, the level of consciousness, pain, and urinary output. The RDAT utilizes a dichotomous (yes/no) assessment approach with 10 items, including activity, respiration, pulse, blood pressure, temperature, oxygen saturation, the level of consciousness, pain, nausea, and surgical bleeding. To be eligible for discharge, patients had to receive a “yes” response for all items [23].

#### 3.1.6. Anesthesia and Perioperative Medicine Service Checklist (SAMPE)

The SAMPE, a checklist for discharge from the recovery room, was recently developed and named after the hospital department where anesthesiologists examined this novel discharge tool [22]. The original SAMPE was introduced in 2013 to address the need for standardized discharge guide from the RR [24]. The vital signs, level of consciousness, and activity items in the first version have scores ranging from 0 to 2. In addition, items measuring oxygen saturation, discomfort, nausea and vomiting, and surgical bleeding were scored on a scale of 0 to 2. The scale has a maximum score of 16 and a cut-off value of 13 [24]. The initial version of the SAMPE was updated in 2015 with the goal of improving adherence and making it easier to use. The most difficult part was determining how to assign points to various items. Therefore, the scale transitioned from point-based evaluation to a dichotomous response (“yes/no”) assessment. Although the eight evaluation items remained the same, the updated checklist now includes detailed descriptions of the items and reference parameters to aid in evaluation [24].

#### 3.1.7. Post-Anesthetic Care Tool (PACT)

Street et al. recently developed the Post-Anesthetic Care Tool (PACT), a new nursing assessment tool designed for use in the postoperative period. The PACT facilitates the communication of a patient’s postoperative experience at different stages and is intended to be integrated into the postanesthetic documentation of a large health service [32].

For patients to be eligible for discharge using the PACT, they must meet the following criteria: (i) no active vomiting; (ii) the last two sets of observations must not fall within a range that necessitates calling the Medical Emergency Team (MET); (iii) ongoing pain medication must be prescribed; (iv) the dermatology level was at least T4 (if applicable); and (v) all surgical concerns must be addressed.

The use of the PACT has been shown to enhance patient assessment and management during the immediate postoperative phase. It improves nurses’ ability to identify and respond to clinical deterioration by ensuring timely physician consultation and preparing the patient for discharge from the RR. Additionally, their findings demonstrated enhanced information sharing amongst nurses related to PACT introduction during clinical handover from the postanesthetic care unit to the ward.

#### 3.1.8. Postoperative Quality Recovery Scale (PQRS)

The Postoperative Quality Recovery Scale (PQRS) is a quick tool that allows recovery assessment in many domains and during various time periods [33]. It requires preoperative evaluations, which serve as the foundation for subsequent evaluations; moreover, the scale reported strong face validity.

It consists of six categories of recovery, each composed of a set of questions: cognitive, nociceptive, emotional, physiological, activities of daily living (ADL), and overall patient perspective. There are findings in nociceptive, emotional, ADL, and overall patient perspectives that can be rated in a categorical manner. Tasks in the cognitive domain were rated based on performance. Based on normative population data, values in the physiological domain were transformed and classified as acceptable, either slightly or significantly outside the ideal ranges. Baseline measurements are essential to use the tool, as the definition of recovery adopted by the PQRS group is “return to baseline values or better”.

#### 3.1.9. Discerning Post-Anesthesia Readiness for Transition (DPART)

The Discerning Post-Anesthesia Readiness for Transition (DPART) is a 16-items instrument developed through collaborative discussions based on professional evidence. The tool is divided into three categories according to patient’s acuity: critical care, in-patient/observation, and outpatient [34]. Items 1 through 16 provide transition criteria specifically for outpatients.

For outpatient discharge at home or outpatient service, higher transition thresholds must be met to ensure patient safety. The previously described evidence-based criteria for items 1 through 13 must also be met by outpatients in addition to the additional requirements for oxygenation and mental alertness. In addition, the patient must be discharged under the supervision of a responsible adult. The DPART tool has undergone content and face validity testing, as well as reliability testing, in both adult and pediatric PACUs.

### 3.2. Considerations on the Retrieved Tools

The Aldrete score and its modified version (MASS) were the first documented scores in the literature for major physiological function assessments. Thus, many scores for discharging patients from the RR were influenced by the Aldrete score, both in the point allocation system and the selection of key assessment items, such as respiration, circulation, and consciousness.

Indeed, Ego et al. analyzed the causes of delays in discharging patients assessed with the Aldrete score, finding that non-clinical reasons were the primary cause in more than 60% the cases [41]. In addition, Awad et al. applied the Aldrete score to patients receiving low doses of intrathecal bupivacaine, seeing it as a tool able to facilitate discharge. In their assessment, they incorporated the type of intrathecal anesthesia and postanesthesia activity as additional criteria for the discharge score [42].

A study comparing time-based discharge (TBD) with score-based discharge was conducted on 100 ASA Physical Status Classification Class I and II patients undergoing minor elective surgery [43], and all patients were discharged at a predetermined time (i.e., 60 minutes after RR admission) according to the TBD in the absence of complications. Simultaneously, patients were evaluated with the MASS and White and Song scores, resulting in 93% of the patients with a MASS score of 9 or higher after 10 min, while the remaining 7% reached the same scores after 20 minutes of stay in the RR. Indeed, it has been demonstrated that the MASS provides a rapid and safe tool for the assessment of patient discharge from the RR while still providing postoperative care [43].

As for the MASS, a prospective randomized study was conducted in 2021 to investigate the safety of assessing the cognitive status of patients receiving desflurane or propofol anesthesia. Sixty patients were enrolled, and their preoperative cognitive status was evaluated using the Digit Symbol Substitution Test (DSST), Stroop Color Test (SCT), and Verbal Learning Test (VLT). The same assessment was performed in the postoperative period in patients who achieved an Aldrete score of ≥9, indicating satisfactory cognitive recovery (except for 10% of patients who still had cognitive dysfunction) [44]. Despite being the oldest scoring system, the modified Aldrete score remains the most used tool in clinical practice. Its distinctive feature is the discharge of patients from the RR to another ward, rather than at home. Mild hypothermia (32–35 °C) is a common complication of surgical procedures that can be managed in in-patient units. Conversely, active bleeding justifies the prolongation of stay in the RR, as observed in other scores such as the PADSS and MPADSS [19]. The absence of this discharge criterion in the MASS may limit a comprehensive evaluation of patients. However, the score continues to be widely used and is supported by numerous studies, even 50 years after its original version was first introduced in clinical practice [45,46,47,48].

REACT was developed to improve the criteria for discharging patients from the RR, incorporating the assessment of body temperature, respiratory rate, and pulse. However, this method has several limitations. Even in its validation study, some patients who met the REACT cut-off score for discharge did not achieve the minimum scores required by other reference standards [23]. Finally, the limitations of this score seem to outweigh its strengths. Notably, REACT does not include an assessment of oxygen saturation, which is a significant drawback. In contrast, the Aldrete score was updated to include this criterion, addressing an initial gap. Additionally, REACT’s inability to be used in the presence of cardiorespiratory complications—common in the recovery room—further limits its applicability [49].

The modified version of the PADSS, the MPADSS, was the first score specifically developed for ambulatory surgery, with the aim of discharging patients directly to their homes on the same day as the surgery. The initial version of the PADSS introduced new items not present in previous scores, such as Aldrete (i.e., surgical bleeding, nausea and vomiting, pain, and fluid balance). The initial PADSS version was validated in a study involving 106 women with breast cancer undergoing surgery [50]. This study found that 85% of patients were discharged within 48 h of surgery based on the PADSS score. Patients who did not achieve a minimum PADSS score of 9 experienced complications such as bleeding, flap necrosis, and surgical wound infection. However, given the controversies surrounding the assessment of intake and output, the score was modified accordingly. In 2013, the MPADSS was used to discharge patients from the RR following colonoscopy procedures under sedation [24]. Discharging patients using the MPADSS was found to be faster than discharge, based on clinical assessment, while maintaining the same level of safety. None of the patients experienced complications requiring hospitalization during follow-up. Mild post-colonoscopy symptoms were reported in 32 patients evaluated using the MPADSS, whereas 57 patients discharged based on clinical evaluation reported such symptoms. Palumbo et al. confirmed that the PADSS is an efficient and safe system for patient discharge [51].

In 2020, a study in France examined the feasibility of performing arthroplasty procedures in an outpatient settings [52]. Twenty-four patients with ASA I and II statuses and without comorbidities underwent the procedures and were discharged home using the modified PADSS. Only two patients did not achieve a PADSS score of at least 9. None of the patients discharged from outpatient settings experienced any complications, and all expressed satisfaction with the functional outcome of the procedure. However, the PADSS score has some limitations because it does not consider the evaluation of oxygen saturation or level of consciousness. Nonetheless, it is considered to be highly conservative and safe. In fact, a 2019 study that used the PADSS to determine the appropriateness of discharging patients home following robot-assisted prostatectomy found that one patient out of 97 achieved a PADSS score ≥ 9 on the day of treatment. Approximately 74% of patients met the discharge criteria on the first day following the procedure [53]. A previous study conducted on women undergoing breast surgery revealed that safe discharge using the PADSS occurred within 48 h after the surgical intervention, rather than within 24 h [24].

In the same year of the PADSS development, Aldrete created the “same day surgery” score [17]. This tool assesses 10 items to determine discharge eligibility from the RR. It was more comprehensive than both the PADSS and its modified version. However, its implementation in clinical practice has not been described. Furthermore, the MPADSS and MASS were found to complement each other because these two scores effectively allowed patients to be discharged on the same day as the procedure for ambulatory surgery under general anesthesia for strabismus correction [54]. The MASS was administered every 15 minutes from the patient’s arrival at the RR until a score equal or higher than 9 was achieved; once this score was reached, the patient could be discharged home. In this study, all the patients were discharged on the same day as the procedure.

The White and Song score was developed for ambulatory surgery and included two additional items: pain and emetic symptoms. As patients were discharged directly to their homes, it was crucial to address the main complications and sources of discomfort. Therefore, in addition to assessing hemodynamics, respiration, oxygenation, the level of consciousness, and motor activity, it was judged vital to address nausea, vomiting, and pain and ensure that they were effectively controlled at the time of discharge [55,56].

The validity and safety of the White and Song scores were demonstrated in a study comparing the use of TBD with MASS and the White and Song scores in patients undergoing minor surgery. The results indicated that 85% of the sample had a score ≥ 12, with a minimum score of 1 for each item in the operating room before entering the RR. These were the same patients who, within 10 min of entering the RR, achieved a score of at least 9 on the MASS and were deemed ready for discharge [43].

A study conducted on patients undergoing surgery under general anesthesia used the White and Song scores to discharge patients directly from the RR to their homes [57]. Of the 50 patients who underwent laparoscopic cholecystectomy, 47 were discharged on the same day. In addition to the White and Song scores, the authors also considered the patient’s ability to tolerate oral fluids, empty the bladder, and walk autonomously [57].

Research carried out in 2003 demonstrated the feasibility of discharging patients from the operating room to a dedicated area called “fast-tracking PACU” and then directly home using the White and Song score [58]. This study included 1380 patients who underwent minor ambulatory surgeries under three types of anesthesia. Upon arrival at the RR, patients were assessed using the White and Song scores. A minimum score of 12 allowed patients to be admitted to the “fast-tracking PACU” area. Patients who did not reach this minimum score were admitted to the hospital following the conventional RR protocol. Among the 952 patients admitted to the fast-tracking area, 88% were discharged within a maximum of 60 min, while the remaining 12% were discharged within a maximum of 100 min. The 428 patients admitted to the RR and subsequently to the day surgery unit required a maximum of 150 min of RR stay before discharge. This emphasizes the possibility of reducing discharge times to home by utilizing a dedicated area (the fast-track area), compared to the time required for discharge via admission to the RR and the day surgery unit. Notably, the White and Song scores considered fewer items than those resulting from the combination of the MASS and MPADSS, which has proven effective in the discharge process of ambulatory surgery [54]. Currently, the White and Song score is primarily used to discharge patients from the RR to the second postanesthesia phase. White and Song suggested that the implementation of their scores could lead to a reduction in hospitalization costs. However, studies conducted at the Department of Anesthesiology of Toronto Western Hospital and the Capital Health Region in Victoria indicated that costs remained unchanged after their use [59,60].

The RDAT was the first instrument to modify the item evaluation system, with dichotomous responses on its 10 items [40]. The RDAT score was compared to the REACT score in two hospitals in southwestern United States [23]. A total of 202 patients were evaluated every 30 minutes by two nurses using both RDAT and REACT. This study demonstrated 100% agreement between nurses when using the RDAT. Furthermore, the RDAT was deemed safe and easy to use. Moreover, comparing the effectiveness of the two scores, REACT showed a lower safety profile because some patients reaching the cut-off for discharge from the RR presented the need to stay longer in the RR for clinical monitoring reasons [23]. A significant advantage of RDAT is its comprehensive nature as it does not require additional information. Furthermore, the use of dichotomous responses promoted a higher level of agreement compared to scores utilizing item points ranging between zero and two [23]. However, the RDAT showed limited flexibility. For example, discharge can be delayed, even when a patient’s condition is clinically stable. Nevertheless, the authors recommended conducting further tests before their widespread adoption [23].

SAMPE is the latest RR score introduced in the scientific literature. The initial version used a classical evaluation system with a zero-to two-range scoring system for individual items. However, the most recent version of this tool uses a dichotomous response (yes/no) to simplify patient discharge and enhance user-friendliness. In a validation study, the SAMPE score was compared to the Aldrete and White and Song scores [22]. A total of 997 patients were assessed using all three scoring systems 90 minutes after a variety of surgical procedures. According to Aldrete et al., 93% of the sample (*n* = 934) met the minimum discharge score, 88% (*n* = 880) met the White and Song criteria, and 73.6% (*n* = 734) met the SAMPE criteria. The authors stated that SAMPE exhibited more conservative characteristics than the other scores because of the narrower dichotomous response system, thus providing increased safety during the discharge process [22,23]. This suggests that SAMPE, being very conservative, could determine the risk related to an increased in-hospital stay duration and its costs. However, both SAMPE and RDAT have similar limitations. The criteria evaluated by SAMPE may be more suitable for ambulatory surgery, as it is rigorous. However, there are currently limited data to completely justify the widespread use of the SAMPE score, most likely due to the necessity for additional research, as this scoring system was first mentioned in the scientific literature in 2022.

Traditional score systems such as the Aldrete and MASS provide a solid basis for assessing the patient. Recently, new scales such as the PACT, PQRS, and DPART have been introduced, which seek to improve and expand existing assessment criteria but are less well known and less used in clinical practice. These newer scores offer more comprehensive and detailed assessments that incorporate a greater variety of parameters to ensure safe and optimal patient recovery.

The PACT scale offers a comprehensive assessment and is designed to provide a detailed and thorough assessment of patient recovery, ensuring that all critical aspects are closely monitored [40]. If we compare newer scales such as the PACT with scores on older scales such as the Aldrete or White and Song scales [14,19], it becomes apparent that the approach is more modern and comprehensive, combining elements of the other scales with a focus on a holistic and detailed approach.

In contrast, the PQRS assesses not only physiological parameters but also emotional, activity, and common symptoms, such as pain [33]. This scale is designed to provide a holistic view of patient recovery, considering both the physical and psychological aspects of postanesthesia recovery. The PQRS scale provides a detailed, multidimensional assessment, making it ideal for the in-depth monitoring of postanesthesia recovery. On the other hand, scales such as Aldrete, MASS, and White and Song, with their simplicity and focus on fundamental physiological parameters, are extremely practical for rapid and immediate assessment in clinical settings where a quick decision on patient safety is needed for transfer or discharge [14,15,19].

Conversely, the DPART is a simple and straightforward tool for recovery assessment and is particularly useful in specific settings where rapid and efficient monitoring is needed by the patient by focusing on essential physiological parameters [32]. The DPART scale is designed for a simple and rapid assessment of patients’ physiological stability after anesthesia, focusing on key parameters such as respiratory rate, pulse rate, blood pressure, and the level of consciousness. It is ideal for settings where a decision is needed quickly for patient safety, but it may leave out important assessments, especially if discharge from the RR involves transferring the patient to an ordinary in-patient setting.

## 4. Discussion

While some facilities use time-based criteria to assess a patient’s readiness for discharge, others use more complete physiological assessment-based criteria or factors, such as pain and vital signs. Despite the availability of recommendations for assessing the discharge readiness of an RR patient, there is no gold standard [60]; thus, we compared the retrieved tools for descriptive purposes only. The present review identified several scores for patient discharge from the recovery room.

In summary, multiple scoring systems have been developed to assess patient discharge readiness based on the RR. In recent years, new scales have emerged to address the need to improve efficient time management and cost-effectiveness, such as White and Song scores. Comparing the different scoring systems, it is apparent that only a few parameters (i.e., blood pressure, respiration, and motor activity) were consistently included in all scales, while other parameters, such as body temperature and surgical bleeding, were less frequently utilized. The limited inclusion of surgical bleeding assessment in some scores may be attributed to the challenge of objective measurements. In fact, where it was included, the assessment was often subjective, based on scales assigning mild, moderate, or severe bleeding, or relying on the changes observed in dressings. This issue introduces subjectivity and complexity to the evaluation of specific items. Currently, the MASS is considered the most reliable scoring system because it considers the major functions affected during the postanesthetic period. The MASS has been extensively used in studies and clinical practice and has paved the way for the development of new scoring systems. Other scores that deviate from this standard have had limited adoption, such as the REACT score, which has been shown to be less safe for patients, or the PADSS, which is rarely used alone [20,23,54]. However, the White and Song scores improved owing to the strengths of the Aldrete score. Although originally designed to assess ambulatory surgery patients, it is currently used as a scoring system for safe discharge from the RR [21].

When determining discharge readiness in the postoperative phase, it is critical to evaluate vital parameters, pain levels, and the occurrence of nausea and/or vomiting. Antiemetic and analgesic therapies are routinely administered even in low-intensity clinical care units. Consequently, both the Aldrete score and White and Song scores were not suitable for ambulatory surgery patients.

Currently, it is not possible to determine the optimal score for patient discharge during ambulatory surgery. The White and Song scores were primarily used for discharge from the first to the second postanesthetic phase, whereas the PADSS was combined with the MASS. Among the compared scores, the “same-day surgery” score assessed the highest number of items and included a unique feature of urinary output, which was not present in other scores, except in the initial version of PADSS. These characteristics contribute to the comprehensiveness of the score, even if at present there is a lack of supportive evidence for this score.

Furthermore, it must be determined whether the use of dichotomous responses is superior to that of point systems [22,23]. The complexity associated with assigning scores between zero and two based on the patients’ clinical conditions has been identified as the main reason for low adherence to the SAMPE scoring system [22]. In contrast, RDAT and SAMPE scores offer an easier approach by utilizing dichotomous responses and providing clearer descriptions of the clinical features to obtain a specific item response [22,23].

To the best of our knowledge, this is the first review to provide an overview of scoring systems that can be used to discharge patients from RRs to surgical wards and, in specific cases, to their homes.

Some limitations should be mentioned. Given the narrative nature of this review, future studies should adopt more systematic approaches, such as a COSMIN review, to ensure a rigorous methodology. The current review lacks a strict and systematic approach, which is its most significant limitation, as relevant studies on the recovery room and PACU may have been overlooked. However, our primary aim was to provide an overview of the topic and the tools identified. To the best of our knowledge, we have included the most-used scoring systems.

Lastly, significant benefits that could increase accuracy and efficiency in healthcare settings include the following: the reduction of compilation time: data entering, and computation processes should be automated; the reduction of calculation errors: the majority of inaccuracies in manual score computation should be eliminated, plus enhanced data exchange and accessibility, as well as digital data archiving and retrieval to enable case reviews and follow-ups, should be implemented. Therefore, healthcare organizations could enhance overall resource optimization and assessment accuracy while also improving service quality and workforce management using automated scoring.

## 5. Conclusions

Identifying formal discharge tools for postoperative patients from the RR has the potential to enhance productivity, prevent complications, and appropriately use resources. Postanesthesia discharge tools have evolved from simple physiological checklists, like the Aldrete score, to more sophisticated systems such as DPART and PACT, which account for different patient acuity levels, safety thresholds, and multidisciplinary care needs.

Indeed, our narrative review discusses several tools, and we found that REACT was inadequate, while RDAT and SAMPE lacked of scientific evidence.

The Aldrete and White and Song scores are currently the preferred options adopted by RR nursing staff because of their safety and widespread use. While the SAMPE scoring system shows promise, it currently lacks of sufficient scientific evidence and requires further validation to become a reliable tool. Finally, to facilitate their practical application, next-generation scores like RDAT and SAMPE should be improved, especially to minimize errors among less-experienced staff.

In conclusion, while simpler tools ensure ease of use, the more recent instruments provide thorough, evidence-based criteria that improve patient safety and enhance communication among healthcare teams. The key considerations moving forward will involve balancing the complexity of assessment tools with practical usability, ensuring that they are both comprehensive and time-efficient.

Surveys and primary design studies on this topic could be helpful in identifying the safest and easiest-to-use scores that would enhance recovery-room nursing and patient safety.

## Figures and Tables

**Table 1:** Summary of the characteristics of discharge scores currently used in RR and PACU.

Score	N. Items	Score Range	Discharge Cut-Off from the RR	Discharge to Hospital Ward or Home	Validity	Strengths	Weaknesses
Aldrete Score System, 1970 [16]	5	0–10	Score ≥ 9	Hospital ward	NR	Evaluation of major physiological functions.	Absence of the item “oxygen saturation”.
Modified Aldrete Score System, 1995 [17]	5	0–10	Score ≥ 9	Hospital ward	NR	Evaluation of major physiological functions. Addition of the item “oxygen saturation”. Safety in clinical practice.	Failure to assess nausea, vomiting, pain, and surgical bleeding.
Same-Day Surgery, 1995 [17]	10	0–20	Score ≥ 18	Hospital ward	NR	/	Lack of literature studies on its use.
PARSAP [31]	8	0–16	Score ≥ 16	Home	NR	The addition of the items “Dressing/operative site, pain, ambulation, fasting/feeding, urine output” improves patient assessment.	Patients are called home for a health check. Outpatient surgery only. Lack of studies supporting its use.
REACT, 1984 [18]	5	0–10	Score ≥ 9	Hospital ward	NR	Inclusion of body temperature.	Failure to evaluate oxygen saturation. Not recommended for use in case of complications. Poor safety in clinical practice.
PADSS, 1995 [19]	5	0–10	Score ≥ 9	Home	Correlation with discharge criteria Pearson’s Correlation Coefficient r = 0.89). The internal consistency reliability of PADSS (alpha = 0.65)	Inclusion of intake/output, nausea, vomiting, pain, and surgical bleeding.	Failure to evaluate oxygen saturation. Controversies in assessing intake/output for discharge to home. Use in conjunction with MASS.
Modified PADSS, 1995 [20]	5	0–10	Score ≥ 9	Home	NR	Inclusion of nausea, vomiting, surgical bleeding.	Failure to evaluate consciousness level, oxygen saturation. Use in conjunction with MASS.
White and Song, 1999 [21]	7	0–14	Score ≥ 12	Home	NR	Inclusion of nausea and vomiting. Safety in its use due to the requirement of a minimum score of 1 for each item.	Insufficient for direct discharge to home. Commonly used in clinical practice for discharge from RR to ward.
RDAT, 2017 [22,23]	10	Yes/No	All answers “yes”	Hospital ward	Content validity index 5.80; inter-rater reliability index 5.10.	Safety in discharge due to evaluation through a dichotomous response. High agreement among evaluators.	Lack of studies supporting its use. Risk of not discharging patients who meet the strict criteria.
SAMPE, 2022 [22,24]	8	Yes/No	All answers “yes”	Hospital ward	Agreement on discharge from RR SAMPE vs. White K 0.69 (95%CI: 0.65–0.74) SAMPE vs. Aldrete K 0.58 (95%CI: 0.53–0.63) Aldrete vs. White K 0.48 (95%CI: 0.39–0.57)	Safety in discharge due to evaluation through a dichotomous response. Detailed description of how the patient should present.	Lack of studies supporting its use. Risk of not discharging patients who meet the strict criteria.
PACT [32]	4	Yes/No	All answers “yes”	Hospital ward	NR	The score is associated with a standardized method for handover between nurses.	Lack of studies supporting its use.
PQRS [33]	6	Recovered/not recovered	All answers “recovered”	Hospital ward	NR	It takes into account each parameter by grouping them into “domains” and re-evaluates them at multiple times during the hospital stay.	Lack of studies supporting its use.
DPART [34]	16	0–16	Score = 3 for discharge to ICU, score = 13 for discharge to hospital ward, score = 16 for discharge to home	Hospital ward or home	Content validity: mean I-CVI, S-CVI/UA, and mean CVR were 1.000 Face validity I-CVI 1; CVR 1 Various degrees of inter-rater reliability used in pediatric and adult PACU	Score also validated for use on the pediatric patient, divided into 3 parts according to the destination of the patient.	The score was compared only with a second score that the experimenting hospital adopted for discharge from RR. Lack of studies supporting its use. Risk of not discharging patients who meet the strict criteria.

Legend. CVI: content validity index; CVR: content validity ratio; DPART: Discerning Post-Anesthesia Readiness for Transition; NR: not reported; PACT: Post-Anesthetic Care Tool; PARSAP: Aldrete’s Expanded Post- Anesthetic Recovery Score for Ambulatory Patients; PQRS: Postoperative Quality Recovery Scale; RDAT: Readiness for Discharge Assessment Tool; REACT: Respiration, Energy, Alertness, Circulation, Temperature; S-CVI/UA: scale content validity index based on universal agreement between raters; SAMPE: Scoring for Ambulatory Patients in the Recovery Room.

**Table 2:** Comparison among the variables included by the different recovery room scores.

	Item									
Score	Activity	Respiratory Rate	Pulse Rate	Blood Pressure	Temperature	Oxygen Saturation	Level of Consciousness	Pain	Nausea/Vomiting	Surgical Bleeding
Aldrete Score System [16]	✓	✓	/	✓	/	✓	✓	/	/	/
MASS [17]	✓	✓	/	✓	/	✓	✓	/	/	/
PARSAP [31]	✓	✓	/	✓	/	/	✓	✓	✓	✓
REACT [18]	✓	✓	✓	✓	✓	/	✓	/	/	/
PADSS [19]	✓	✓	✓	/	/	/	/	/	✓	✓
MPADSS [20]	✓	✓	✓	✓	✓	/	/	✓	✓	✓
White and Song [21]	✓	✓	/	✓	/	✓	✓	✓	✓	/
RDAT [22,23]	✓	✓	✓	✓	✓	✓	✓	✓	✓	✓
SAMPE [22,24]	✓	✓	✓	✓	/	✓	✓	✓	✓	✓
PACT [32]	/	/	/	/	✓	/	/	✓	✓	✓
PQRS [33]	✓	✓	✓	✓	✓	✓	✓	✓	✓	/
DPART [34]	✓	✓	/	✓	✓	✓	✓	✓	✓	✓

Legend. REACT: Respiration, Energy, Alertness, Circulation, Temperature; RDAT: Readiness for Discharge Assessment Tool; SAMPE: Scoring for Ambulatory Patients in the Recovery Room; PARSAP: Aldrete’s Expanded Post-Anesthetic Recovery Score for Ambulatory Patients; PACT: Post-Anesthetic Care Tool; PQRS: Postoperative Quality Recovery Scale; DPART: Discerning Post-Anesthesia Readiness for Transition; ✓: available; /: not available.

## Data Availability

Not applicable.

## Supplementary Materials

The following supporting information can be downloaded at https://www.mdpi.com/article/10.3390/nursrep14040205/s1. Table S1: SHC-21-17-Reporting Checklist; File S2: search strategy for each database.

## Public Involvement Statement

No public involvement in any aspect of this research.

## Guidelines and Standards Statement

This manuscript was drafted against the SHC-21-17 reporting checklist for narrative reviews. See [29].
