# Supplementary material for: A Comparison among Score Systems for Discharging Patients from Recovery Rooms: A Narrative Review
Source: Nurs Rep. 2024 Oct 6;14(4):2777–94. doi: 10.3390/nursrep14040205 (PMC11503295; doi:10.3390/nursrep14040205)
Supplement: Supplementary file 1 [file nursrep-14-00205-s001.zip › nursrep-3065474-Supplementary File S2.pdf]

Database	Search string	Results
PubMed/MEDLINE<br><br>Filters:<br>- Languages: English	("Recovery Room"[Mesh] OR "Postanesthesia Nursing/organization and administration"[Mesh] OR "Anesthesia Recovery Period"[Mesh] OR "Postoperative Care"[Mesh]) AND ("Patient Discharge"[Mesh] OR "Hospital to Home Transition"[Mesh] OR "Patient Handoff"[Mesh] OR "Physical Examination"[Mesh] OR "Nursing Assessment"[Mesh])) AND (scor* OR checklist OR measur* OR criteria OR scal*)	3194
Cumulative Index to Nursing and Allied Health Literature (CINAHL)<br><br>Filters:<br>- Languages: English	((MH "Post Anesthesia Care Units") OR (MH "Anesthesia Recovery") OR (MH "Postoperative Care")) AND ((MH "Patient Discharge") OR (MH "Hospital to Home Transition") OR (MH "Hand Off (Patient Safety)") OR (MH "Physical Examination") OR (MH "Nursing Assessment")) AND (scor* OR checklist OR measur* OR criteria OR scal*)	449
Embase<br><br>Filters:<br>- Articles<br>- Sources: Embase	(('recovery room'/exp OR 'postanesthesia care'/exp OR 'anesthetic recovery'/exp OR 'postoperative care'/exp) AND ('hospital discharge'/exp OR 'hospital to home transition'/exp OR 'clinical handover'/exp OR 'physical examination'/exp OR 'nursing assessment'/exp)) AND (scor* OR checklist OR measur* OR criteria OR scal*)	2736
