# Supplementary material for: A Comparison among Score Systems for Discharging Patients from Recovery Rooms: A Narrative Review
Source: Nurs Rep. 2024 Oct 6;14(4):2777–94. doi: 10.3390/nursrep14040205 (PMC11503295; doi:10.3390/nursrep14040205)
Supplement: Supplementary file 1 [file nursrep-14-00205-s001.zip › nursrep-3065474-Supplementary Table S1.pdf]

Narrative Review Checklist

Section/Topic	Item No	Item	Reported on Page Number/Line Number	Reported on Section/Paragraph
**TITLE**				
Title	1	Identify the report as a Narrative Review or Literature Review.	1	TITLE
**ABSTRACT**				
Structured summary	2	Provide a structured summary with the subsections as: objective, background, methods, conclusion (1).	1	ABSTRACT
**INTRODUCTION**				
Rationale/background	3	Describe the rationale for the review in the context of what is already known.	2-3	INTRODUCTION
Objectives	4	Specify the key question(s) identified for the review topic.	3	INTRODUCTION
**METHODS**				
Research selection	5	Specify the process for identifying the literature search (eg, years considered, language, publication status, study design, and databases of coverage).	3	MATERIALS AND METHODS
**DISCUSSION/SUMMARY**				
Narrative	6	Discuss: 1) research reviewed including fundamental or key findings, 2) limitations and/or quality of research reviewed, and 3) need for future research.	4-12	RESULTS/DISCUSSION
Summary	7	Provide an overall interpretation of the narrative review in the context of clinical practice for health professionals, policy development and implementation, or future research.	4-12	RESULTS/DISCUSSION

Reference

1. Green BN, Johnson CD, Adams A. Writing narrative literature reviews for peer-reviewed journals: secrets of the trade. J Sports Chiropr Rehabil 2001;15:5–19.

Article information: https://dx.doi.org/10.21037/shc-21-17
*As the checklist was provided upon initial submission, the page number/line number reported may be changed due to copyediting and may not be referable in the published version. In this case, the section/paragraph may be used as an alternative reference.
